# Factors Associated with Disability in Rural Bangladesh: Bangladesh Population-Based Diabetes and Eye Study (BPDES)

**DOI:** 10.1371/journal.pone.0165625

**Published:** 2016-12-09

**Authors:** Fakir M. Amirul Islam, Jahar L. Bhowmik, Silvia Z. Islam, Andre M. N. Renzaho, Janet E. Hiller

**Affiliations:** 1 Department of Statistics, Data Science and Epidemiology, Faculty of Health, Arts and Design, Swinburne University of Technology, Hawthorn campus, Hawthorn, Victoria, Australia; 2 Organization for Rural Community Development, Dariapur, Narail, Bangladesh; 3 School of Economics, Finance and Marketing, RMIT University, Melbourne, Victoria, Australia; 4 Humanitarian and Development Studies, School of Social Science and Psychology, Western Sydney University, Penrith, New South Wales, Australia; 5 Department of Epidemiology and Preventive Medicine, Monash University, Melbourne, Australia; 6 School of Health Science, Faculty of Health, Arts and Design, Swinburne University of Technology, Hawthorn campus, Hawthorn, Victoria, Australia; 7 School of Population Health, University of Adelaide, Adelaide, Australia; Universita degli Studi di Perugia, ITALY

## Abstract

**Background:**

To assess factors associated with disability in a rural district of Bangladesh.

**Methods:**

Using a population-based systematic sampling technique, data were collected from 3104 adults aged ≥ 30 years from the Banshgram union of Narail district. Data collected included an interviewer administered questionnaire to report physical disabilities including impairment that prevents engagement with paid work, visual, hearing, and mobility as well as mental disabilities. Socio-demographic and anthropometric factors including educational attainment and body mass index, as well as clinical factors such as blood pressure, and fasting blood glucose were also collected. Binary and multinomial logistic regression techniques were used to explore the association of various socio-demographic and clinical factors with disability.

**Results:**

The mean (SD), minimum and maximum ages of the participants were 51 (12), 30 and 89 years. Of total participants, 65% were female. The prevalence of disability varied from 29.1% for visual impairment (highest) to 16.5% for hearing, 14.7% for movement difficulties and 1.6% (lowest) for any other disability that prevented engagement with paid work. Overall, the prevalence of a single disability was 28.6% and that of two or more disabilities was 14.7%. Older age, gender (female), lower socio-economic status (SES), and hypertension were associated with a higher prevalence of most of the disability components. The prevalence of hearing problems (24.5% vs. 13.3%, p<0.001) and movement difficulties (24.9% vs. 13.0%, p<0.001) was significantly higher among lower-income participants than their higher-income counterparts after controlling for age. Prevalence of visual impairment (54.6% vs. 9.2%, p<0.001), hearing (32.2% vs. 6.7%, p<0.001) and movement difficulties (29.2% vs. 5.5%, p<0.001) were significantly higher in people of aged 60 years or older than those aged 30–34 years. After multivariate adjustment, the prevalence of single disability (prevalence risk ratio [PRR] 1.25, 95% CI: 1.09–1.42, p<0.001), and multiple disabilities (PRR 1.41, 95% CI 1.14–1.73, p<0.001) was higher among females than males. The prevalence of single disability and multiple disabilities was respectively 21% (PRR 1.21, 95% CI: 1.02–1.42, p<0.001) and 88% (PRR 1.88, 95% CI: 1.38–2.54, p<0.001) higher among participants with low educational attainment (primary level or less) than those with at least a secondary level of education.

**Conclusions:**

In rural Bangladesh, the prevalence of disability is high. Public health programs should target those of low SES, older age, and female participants and aim to provide necessary supports in order to bridge disability-related inequities.

## Introduction

The 2011 World Health Organization (WHO) report estimated that there were more than 1 billion people living with some form of disability worldwide, with nearly 200 million facing considerable difficulties in functioning and significant proportion of them reside in developing countries [1]. Available estimates suggest that the world prevalence (15%) of disability is expected to increase with the growing ageing population, as disability is consistently associated with older age [2, 3].

One of the most significant studies into disabilities, with a total sample of 21, 8737 respondents, investigated the prevalence of self-reported disability in 49 countries, of which 33 were low and middle income countries (LMICs) [4]. The age and sex adjusted prevalence of disability in LMICs was 15.1% (with 95% confidence interval (CI) of 13.7–18.8%) which was significantly higher than that observed in higher income countries (10.8%; 95%CI: 6.5–13.4%). The highest prevalence of disability was reported in Bangladesh (32%), followed by India (24.9%) [4]. It is worth noting that the prevalence of disability in most LMICs has been found to be higher in rural areas than in urban areas [4–6].

It has been consistently found that increased age is associated with chronic diseases such as hypertension, diabetes, visual impairment and mental health disorders [7–11]. Some of the conditions such as visual impairment and mental health disorders are closely related to disability [12–15]. With Bangladesh experiencing an exponential increase of the aging population and predominantly made of rural populations (78% of the total population live in the rural area) [16], the number of people with disability is likely to increase in the next two decades [2, 3, 17].

There are different estimates of the prevalence of disability in Bangladesh. Reported prevalence ranges from a minimum of 0.5% to a maximum of 31.9% [1–3, 17, 18]. Older age, lower socio-economic status and lower level of education have been found to be consistently associated with a higher prevalence of disability in Bangladesh [2, 3]. However, no studies have reported the association between disability and objectively measured fasting glucose (type 2 diabetes) and blood pressure (hypertension) among the general population. Both Type 2 diabetes and hypertension are associated with disability in rural Bangladesh and remain the leading cause of heart disease, stroke, and visual impairment [19, 20].

Using data from the Bangladesh population-based diabetes and eye study (BPDES) [21], the current study aimed to provide a further estimate of prevalence of disability and associated risk factors amongst adults aged 30 years and older in a rural district in Bangladesh.

## Materials and Methods

### Study sample

The BPDES was initiated in the Banshgram Union of Narail District in 2012–2013 and has been ongoing since then. This paper is based on data obtained in the first phase of the project. The first phase of the BPDES targeted adults aged between 30–89 years to determine their knowledge, attitudes, and practice about diabetes and common eye diseases, measuring fasting blood glucose to estimate known and undiagnosed type 2 diabetes [11] and blood pressure to estimate known and undiagnosed hypertension [7] and information on disability. The study location is approximately 200 km southwest of the capital city of Dhaka, with eligible participants of approximately 5,500 in the Banshgram Union [22]. Narail is considered to be a typical rural district, being neither in a remote area nor in close proximity to metropolitan Dhaka. The similarity of study participants with the population at national level has been reported elsewhere [11, 21]. The sample size was based on the 2012 prevalence of type 2 diabetes (6.3%) among adults in Bangladesh, as estimated by the International Diabetes Federation’s Diabetes Atlas [23]. The estimated total sample was involved 3,104. The sample size had 90% statistical power at a significance level of 0.05 to detect the prevalence of the following conditions: 28% with 95% CIs of 4 percentage points for visual impairment, 14.5% with 95% CIs of 3 percentage points for hearing problem or difficulties of movement, 1.6% with 95% CIs of 0.8 percentage points for inability to engage in paid work.

### Data collection

A population-based systematic sampling technique was used by selecting every second household in each of 18 villages in the Union, starting from the far east corner of each village. Data were collected by a team of four members. Prior to the commencement of the survey, all team members participated in an intensive two day training program in Narail. The training covered the rationale for the study, and all procedures and potential difficulties of data collection including anonymity and confidentiality. A dry run was carried out over a single-day to give enumerators a chance to familiarize themselves with all study procedures and instruments.

Participants were required to have two visits over two days to complete data collection. The first visit involved a face-to-face interview at each participant’s residence to collect data on disability, knowledge, attitudes towards and management of Type 2 diabetes, common eye diseases, and socio-demographic factors including level of education and socio-economic status (SES). The interviewers informed the participants to attend the nearest community center or school next day morning to undertake clinical examination to obtain data on blood pressure, fasting capillary glucose and other anthropometric measures Exclusion criteria were those younger than 30 years, and those who were too unwell to attend the center for clinical examination on the second visit. None of the participants who were approached and met our inclusion criteria refused to participate on the first visit at home. However, on the second visit on next day, about 15% of participants interviewed on the first visit failed to show up for the second visit (with a participation rate of 85%). Selection of the study location, recruitment and data collection related to the current study have been reported in previous publications [11, 21].

### Ethics approval and consent processes

The study adhered to the set of ethical principles outlined in the Declaration of Helsinki and was approved by the Bangladesh Medical Research Council’s Human Research Ethics Committee (Reference: BMRC/NREC/2010-2013/68). The purpose of the study was fully explained to potential participants and written consent was obtained from those who were able to sign, with finger prints obtained from those who were unable to sign (47%). In case of finger print consent, the data collector provided a counter signature for the participants. Participants were informed of their right to withdraw from the study at any stage if they desired.

### Disability measuring tool and the outcome variables

Disability data were collected using a modified version of a validated disability measurement questionnaire which was developed for a rapid assessment of disability (RAD) in rural Bangladesh and it was validated in Fiji [24]. The outcome variables were five self-reported items related to disability: (i) inability to engage in paid work, (ii) visual impairment, (iii) hearing difficulties, (iv) difficulties in movement, and (v) mental health disabilities. Participants were asked to rate the frequency of difficulty as ‘not at all’, ‘some of the time’, ‘most of the time’, or ‘all of the time’ for each of the components in last three months preceding the survey. We added a real life example such as “did you have any problems with reading books or papers or estimating how much oil you needed for your cooking”?. Responses were recorded as binary variables with possible values of 0 for “not at all” or “some of the time”, and 1 for ‘most of the time’, or ‘all of the time’. The questionnaire was developed based on the disability measuring tool developed by Washington group [25]. The five items were summed to produce a total variable of “summed scores”, with a minimum of 0 for no disability and a maximum of 5 for five different disabilities. For ease of interpretation, the total variable was categorised as follows: 0 for no disability, 1 for single disability and 2 for two or more disabilities.

### Exposure variables

Demographic details (age, gender), life style factors (tobacco use in terms of smoking or chewing any tobacco product), level of education and SES were obtained. The level of education was categorized as no schooling, primary school education (grade 1 to 5), secondary school education (grade 6 to 10) and secondary school certificate (SSC) (O level equivalent) or above. SES was assessed according to Cheng et al. [26] asking participants the following question: "Over the last twelve months, in terms of household food consumption, how would you classify your socio-economic status?", with possible responses being: (i) insufficient funds for the whole year, (ii) insufficient funds for some of the period, (iii) neither deficit nor surplus (balance) and (iv) sufficient funds most of the time. Anthropometric measurements included height, weight and waist circumference, while data on blood pressure and fasting blood glucose were also obtained. Capillary fasting blood glucose (CFBG) was collected using point-of-care (POC) capillary blood and processed using Accu-Check Inform II (Roche Diagnostics, Australia) which is plasma-calibrated. Blood pressure was measured from the right arm with the person sitting upright, and a further measurement was taken following a period of at least 5 minutes rest. Blood pressure data were collected using a calibrated Omron Premium Blood Pressure Monitor Device (BPMD), which is reported to produce digital and accurate reading utilizing the dual check calibration system. The two readings were averaged for systolic and diastolic blood pressure, separately. Hypertension was defined as either self-report of using medication for hypertension or following direct measurement of blood pressure. Participants were considered to have hypertension if (a) they report to be taking blood pressure lowering medication, (b) recorded a systolic blood pressure ≥140 mmHg and/or diastolic blood pressure ≥90 mmHg. Collection of fasting blood glucose and measurement of blood pressure were reported previously [7, 11].

### Statistical analysis

Participants’ characteristics including age, gender, level of education and SES were summarized using descriptive statistics. The prevalence of disability components was examined with each of the socio-demographic characteristics, type 2 diabetes, hypertension, quartile of BMI and both systolic and diastolic blood pressure, using Chi-square tests. The prevalence of disability components, single and multiple disabilities was standardized based on age specific total population in Bangladesh according to the 2011 Bangladesh Bureau of Statistics (BBS)’s national survey report [18]. The age-specific prevalence for disability was calculated using the population size in different age groups at the national level as the age-specific weight. The direct standardized method *[Σ (r*_*i*_*×P*_*i*_*)/ΣP*_*i*_, where *r*_*i*_ is the prevalence of disability in age group *i* and *P*_*i*_ is the population size in *i*th age-group] was used for calculation the age-standardized prevalence. Binary logistic regression techniques were used to estimate the prevalence rate ratio (PRR) and 95% confidence intervals (CI) for disability items in association with presence versus absence of categorical variables (e.g., hypertension present or absent) or quartiles of continuous variables (e.g., BMI). All models were adjusted for age, diabetes status, level of education, and BMI. A multinomial logistic regression technique was used to determine the PRR of single and multiple disability compared to no disability for different exposure variables. The statistical software SPSS version 23 (IBM SPSS, Armonk, NY, USA) was used for the analysis. However, the SPSS does not directly compute prevalence risk ratio and thus a conversion of odds ratio to prevalence rate ratio was used as per Wang [27].

## Results

Of 3104 participants, 7.7% were aged 30–35 years, and 14.7% were above or equal to 65 years of age, 65% were female, 47% had no schooling, 7.3% had school secondary certificate or above, 14% had insufficient funds most of the time, 7.3% had type 2 diabetes, 40% had either known or newly diagnosed hypertension (Table 1).

**Table 1:** Socio-demographic and clinical factors of the study sample (N = 3104).

		Number	%
Gender	Female	2032	65.5
	Male	1072	34.5
Age in years	Less than 35	238	7.7
	35–44	878	28.3
	45–54	942	30.3
	55–64	590	19.0
	Above or equal 65	456	14.7
Level of education	No education	1462	47.1
	Primary (1–5)	921	29.7
	Secondary (6–10)	495	15.9
	School Secondary Certificate or above	226	7.3
Socio-economic status	Insufficient funds all the time	423	13.7
	Insufficient funds some of the time	1077	34.9
	Balance	1320	42.7
	Sufficient funds most of the time	268	8.7
Smoking or use of any tobacco	No	1883	60.8
	Yes	1214	39.2
Hypertension	Normal	1854	59.9
	known and newly diagnosed	1242	40.1
Type 2 diabetes	Normal or Impaired fasting glucose	2873	92.8
	Diabetes	222	7.2
Body mass index (kg/m^2^) in quartile	Less than 19.02	775	25.0
	19.02–21.29	775	25.0
	21.30–24.25	778	25.1
	Above or 24.26	776	25.0

In the total sample, the crude (population age standardized) prevalence of the various forms of disability was: 1.6 (1.5)% for inability to engage in paid work, 29.1 (25.8)% for visual impairment, 16.5 (14.1)% for hearing difficulty, 14.7 (12.8)% for difficulties in movement, 28.6 (26.4)% for single disability and 14.7 (12.3)% for multiple disabilities. One percent (n = 30) of participants presented with mental disability, but this did not vary by gender (1.1% in females vs. 0.7% in males, p = 0.36) or any other socio-demographic characteristics, except the level of education. That is, participants with low educational attainment (no schooling) had a higher prevalence of mental disability than those with at least SSC or above (1.4% vs. 0.2%, p = 0.04). No further analysis was conducted for mental disability due to the small prevalence of this outcome variable.

After multivariate analyses, adjusting for age, gender, level of education, BMI, type 2 diabetes status and systolic BP, a range of independent variables were found to be associated with a higher prevalence of disabilities. Visual impairment was associated with older age, female gender, and hypertension or higher systolic blood pressure. Hearing difficulties were associated with older age, female gender, hypertension or higher systolic blood pressure, low SES, and smoking. Movement difficulties were associated with older age, female gender, hypertension or higher systolic blood pressure, low SES, smoking, and type 2 diabetes. Overall, the prevalence of visual impairment, hearing difficulties, and movement difficulties was respectively 35% [adjusted prevalence rate ratio (aPRR) 1.35, 95% confidence interval (CI): 1.19–1.52)], 51% (aPRR 1.51, 95% CI: 1.25–1.81), and 53% (aPRR 1.53, 95% CI: 1.25–1.84) higher among females than males. Compared to no smokers, the prevalence of hearing difficulties 26% (aPRR 1.26, 95% CI: 1.05–1.50) and movement difficulties 20% (aPRR 1.20, 95% CI: 1.00–1.45) were higher among smokers (Table 2). However, no significant associations were found for the inability to engage in paid work (results are not shown in Table 2).

**Table 2:** Prevalence and the associations of socio-demographic and clinical factors with three disability components.

		Visual impairment			Hearing problem			Difficulties in movement		
Characteristics	No at risk	N	%	PRR (95% CI)*	N	%	PRR (95% CI)*	n	%	PRR (95% CI)*
Age (years):										
<35	238	22	9.2	1.0	16	6.7	1.0	13	5.5	1.0
35–44	878	129	14.7	1.38 (0.89, 2.08)	78	8.9	1.18 (0.69, 2)	70	8.0	1.18(0.67,2.01)
45–54	942	265	28.1	2.69 (1.85, 3.76)	129	13.7	1.79 (1.07, 2.9)	135	14.3	1.78(1.05,2.93)
55–64	590	230	39.0	3.67 (2.59, 4.94)	124	21.0	2.77 (1.68, 4.35)	99	16.8	2.79(1.66,4.49)
65 and above	456	249	54.6	5.31 (3.97, 6.67)	147	32.2	4.53 (2.88, 6.59)	133	29.2	4.62(2.85,6.97)
Total	3104	902	29.1		512	16.5		456	14.7	
†Gender	3104									
Male	1072	251	23.4	1.0	135	12.6	1.0	118	11.0	1.0
Female	2032	651	32.2	1.35 (1.19, 1.52)	376	18.5	1.51 (1.25, 1.81)	338	16.6	1.53(1.25,1.84)
†Education level	3104									
No education	1462	480	32.8	1.22 (0.88, 1.62)	261	17.9	1.08 (0.67, 1.75)	226	15.5	0.91(0.57,1.43)
Primary (1–5)	921	260	28.3	1.06 (0.76, 1.44)	155	16.8	1.09 (0.67, 1.78)	150	16.3	0.97(0.61,1.52)
Secondary (6–10)	495	112	22.6	0.83 (0.57, 1.18)	67	13.5	0.88 (0.52, 1.48)	61	12.3	0.79(0.47,1.3)
SSC or above	226	54	23.7	1.0	31	13.6	1.0	23	10.1	1.0
†SES	3088									
Insufficient funds all the time	423	113	26.6	0.94 (0.71, 1.24)	104	24.5	1.73 (1.2, 2.4)	105	24.9	1.73(1.2,2.41)
Insufficient funds some of the time	1077	368	34.1	1.23 (0.97, 1.52)	199	18.5	1.31 (0.91, 1.82)	172	16.0	1.32(0.92,1.83)
Balance	1320	355	26.9	0.92 (0.72, 1.18)	176	13.4	0.89 (0.61, 1.27)	146	11.0	0.89(0.61,1.28)
Sufficient funds most of the time	268	67	25.1	1.0	36	13.3	1.0	35	13.0	1.0
†Hypertension	3096									
Normal	1854	495	26.7	1.0	289	15.6	1.0	248	13.4	1.0
known and newly diagnosed	1242	407	32.8	1.28 (1.13, 1.45)	223	18.0	1.21 (1.01, 1.45)	207	16.7	1.31(0.89,1.86)
†Diabetes status	3095									
Normal	2710	786	29.0	1.0	453	16.7	1.0	387	14.3	1.0
IFG	163	52	31.8	1.15 (0.89, 1.46)	24	14.6	0.89 (0.58, 1.32)	26	16.0	1.13(0.76,1.65)
Diabetes	222	64	29.1	1.04 (0.82, 1.29)	36	16.1	0.98 (0.7, 1.35)	45	20.3	1.46(1.08,1.94)
†Smoking status										
Never smoked	1883	543	28.9	1.0	289	15.3	1.0	264	14.0	1.0
Ever smoked	1214	361	29.7	1.05 (0.92, 1.19)	223	18.4	1.26 (1.05, 1.5)	195	16.0	1.20(1.0,1.45)
†BMI	3104									
Less than 19.02	775	255	32.9	1.28 (1.07, 1.5)	128	16.5	1.18 (0.9, 1.52)	105	13.6	1.18(0.91,1.51)
19.02 to 21.29	775	223	28.8	1.08 (0.9, 1.29)	146	18.8	1.31 (1.01, 1.66)	110	14.2	1.31(1.03,1.65)
21.30 to 24.25	778	214	27.5	1.01 (0.83, 1.21)	125	16.1	1.11 (0.85, 1.43)	112	14.5	1.12(0.86,1.42)
24.26 and above	776	212	27.3	1.0	115	14.8	1.0	130	16.7	1.0
†SBP quartile	3096									
Q1, <126mmHg	1364	331	24.3	1.0	211	15.4	1.0	171	12.5	1.0
Q2:126.0–140 mmHg	658	195	29.6	1.34 (1.13, 1.58)	108	16.5	1.17 (0.91, 1.47)	105	15.9	1.44(1.11,1.83)
Q3:140.01-160mmHg	599	189	31.6	1.44 (1.21, 1.69)	97	16.2	1.1 (0.86, 1.4)	98	16.3	1.43(1.11,1.83)
Q4: ≥160mmHg	475	188	39.5	1.77 (1.5, 2.07)	96	20.2	1.4 (1.09, 1.78)	83	17.5	1.52(1.17,1.96)

The list of variable which were checked and found no significant associations: diastolic blood pressure

*Prevalence Risk Ratio (95% Confidence Interval (CI)) adjusted for variables included in the model;

^†^ the prevalence was adjusted for age and gender (gender was adjusted for age only)

Results from multinomial logistic regression models found that participants aged 45 years or older had a significantly higher prevalence of a single disability (aPRR 1.99, 95% CI: 1.74–2.24) and multiple disabilities (aPRR 4.53, 95% CI: 3.48–5.80) than those aged younger than 45 years. Participants with primary education or less had a significantly higher prevalence of single disability (aPRR 1.21, 95% CI: 1.02–1.42) and multiple disabilities (aPRR 1.88, 95% CI: 1.38–2.54), than those with secondary education or higher. Participants with hypertension had a significantly higher prevalence of single disability (aPRR 1.27, 95% CI: 1.11–1.43) and multiple disabilities (aPRR 1.83, 95% CI: 1.50–2.21), than those with normal blood pressure. The prevalence of single disability (aPRR 1.25, 95% CI: 1.09–1.42) and multiple disabilities (aPRR 1.41, 95% CI: 1.14–1.73) was significantly higher among females than males. Type 2 diabetes status did not show any significant associations with single disability or multiple disabilities (Table 3).

**Table 3:** Association of socio-demographic and other characteristics with single and multiple disabilities.

		No disability, N = 1761 (56.7%)		Single disability, N = 887(28.6%)		2 or more disabilities, N = 456 (14.7%)		P for trend
	No at risk	n (%)		n (%)	PRR (95% CI)*	n (%)	PRR (95% CI)*	
Age, 30–44 years	1116	838 (75.1)		222 (19.9)		56 (5)		
≥45 years	1988	923 (46.4)	1.0	665 (33.5)	1.99 (1.74, 2.24)	400 (20.1)	4.53 (3.48, 5.80)	<0.001
Gender, Male	1072	633 (59.0)		293 (27.3)		146 (13.6)		
Female	2032	1128 (55.5)	1.0	594 (29.2)	1.25 (1.09, 1.42)	310 (15.3)	1.41 (1.14, 1.73)	<0.001
Education secondary or above	721	498 (69.1)		170 (23.6)		53 (7.4)		
No education or primary	2383	768 (51.2)	1.0	717 (30.1)	1.21 (1.02, 1.42)	403 (16.9)	1.88 (1.38, 2.54)	<0.001
SES, balance or surplus	1588	986 (62.1)		428 (27)		174 (11)		
Had insufficient funds some or all of the time	1500	768 (51.2)	1.0	452 (30.1)	1.23 (1.08, 1.38)	280 (18.7)	1.87 (1.53, 2.25)	<0.001
Hypertension, absent	1854	1166 (62.9)		488 (26.3)		200 (10.8)		
Present	1242	592 (47.7)	1.0	397 (32)	1.27 (1.11, 1.43)	253 (20.4)	1.83 (1.5, 2.21)	<0.001
Normal or Impaired fasting glucose	2873	1635 (56.9)		826 (28.8)		412 (14.3)		
Diabetes	222	121 (54.5)	1.0	57 (25.7)	0.90 (0.68, 1.17)	44 (19.8)	1.37 (0.97, 1.90)	0.06
BMI 4^th^ Quartile	776	461 (59.4)		221 (28.5)		94 (12.1)		
1^st^ quartile	775	408 (52.6)	1.0	232 (29.9)	1.08 (0.90, 1.27)	135 (17.4)	1.45 (1.08, 1.91)	0.04

*Prevalence Risk Ratio (95% Confidence Interval (CI)) adjusted for variables included in the model

## Discussion

Our study reporting the prevalence of different types of disabilities and their associations with socio-demographic, anthropometric and clinical factors in a typical rural area in Bangladesh addresses a significant gap in the literature. Our findings on the prevalence of disability are consistent with those reported by studies in Bangladesh [1, 2] and neighboring India [4] and are expected to be similar to the prevalence reported in other developing or middle income countries [5, 28]. For example, the prevalence of visual impairment (29.1%), hearing (16.5%) and movement disabilities (14.7%) observed in our study was comparable to the prevalence of disability subtypes reported by Rashed et al. [2]. Rashed et al. reported that 10.4% of people aged 30 years or above had a disability. Amongst the 10.4% with disability, 42.2% had visual impairment, 25.8% had hearing problems and 24.4% had physical disability. Sightsavers International carried out a national survey in 2003 among adults aged 30 years or older and reported that 25.9% of the study population suffered from any sort of visual impairment [29]. Mitra and Sambamoorthi reported a prevalence of disability of 22% among adults in Bangladesh [30] whereas Marella et al [3] reported a prevalence of 10.5%. The lower prevalence of disability reported by Marella and colleagues can be attributed to the fact that participants in their study were aged 18 years or older versus 30 years or older included in our study, with older age consistently associated with a higher prevalence of disability [2, 3, 29–31].

We found that factors consistently associated with most of the disability components in this sample were older age, female gender, lower educational level, lower SES and hypertension. Our finding of a higher prevalence of disability among females than males is consistent with previous studies [3, 31, 32]. This pattern could be explained by a variety of factors, including the fact that females in low income countries are more likely to have poor health and functional disabilities [33], to have low educational attainment and hence making them less knowledgeable about risk factors and management of disease and differential access to care, especially if the care facilities are far away from their residence [11, 34, 35]. In addition, women continue to be victims of physical violence or domestic abuse by their partners, relatives or neighbors and this may have an impact on the prevalence of disability. A study conducted into the experience of 226 disabled women residing in different districts in Bangladesh found that 84% of them had suffered physical and psychological problems due to at least one form of violence including emotional abuse and physical, verbal or sexual violence from their partners during their lifetime [36].

Our findings of a higher prevalence of disability among participants with low educational attainment are consistent with other studies conducted in Bangladesh and India [2, 37]. For example, Rashed et al.[3] reported a prevalence of disability that was five times higher among participants with no education compared to those who had at least secondary level of education. The study also found an inverse association between SES and disability [2]. Marella et al. reported that, compared to people in the richest quintile of SES, people in the poorest quintile had almost double the prevalence of disability [3]. This finding is almost identical to our finding of double the prevalence of hearing and movement difficulties in people who had insufficient funds most of the time compared to those who had adequate funds in most of the time of the year. Our finding of the inverse association between educational attainment and the number of disabilities are consistent with those reported by Rashed et al. [2]. Marella et al. [3] also reported an inverse relationship between educational attainment and disability though it was not statistically significant. The lack of significant association in Marella’s study may be due to the different age and education distributions in their study compared to this study. Our study participants were 30 years or older of whom 47% had no education, compared to Marella’s study with participants of age 18 years or older of whom 37% had no education [3].

An additional finding from our study concerned the impact of cardiovascular disease on disability in rural communities, with an increasing prevalence of visual impairment associated with hypertension. After multivariate adjustment, it was found that hearing difficulty was associated with hypertension as well as smoking, and the movement difficulty was associated with diabetes and higher blood pressure. These findings are consistent with previous studies conducted in Bangladesh, South East Asia, and in other developed countries [17, 38].

Our study has several limitations. Firstly, we selected participants of aged 30 years or older, where most of the other studies have included participants 18 years or older. The selection of this age range excluded a potentially healthy sub-sample and is highly likely to lead to a higher prevalence of disability. Secondly, the female participation was higher and the prevalence of disability was also higher in females. This might cause an overestimate of the overall prevalence of disability though adjustment for gender is expected to diminish the overestimate problem. Most of the related studies have reported a higher prevalence of disability among females and thus our findings might be consistent with the other studies. Thirdly, we used self-reported disabilities, as opposed to diagnosed disability with the possibility of these being reporting error in the data. Fourthly, for practical reasons we did not use WHODAS 2.0, which is the most comprehensive tool to assess disability. WHODAS 2.0 is a generic assessment instrument developed by WHO to provide a standardized method for measuring health and disability across cultures. WHODAS 2.0 was developed from a widespread set of International Classification of Functioning, Disability and Health (ICF) items which are adequately trustworthy and thoughtful to measure the difference made a given intervention [39]. Therefore, our findings need to be interpreted with caution. However, we used a shorter validated questionnaire to measure disability based on the Washington Group Short Set of Questions on Disability [25]. Finally, the study was conducted in only one area of rural Bangladesh. Whilst it is representative of the situation in Banshgram, the findings need to be extrapolated with caution to other rural parts of Bangladesh. Notwithstanding these limitations, our findings are important given the increasing life expectancy in Bangladesh [17, 40] and of the focus on the rural population in Bangladesh [18, 41]. Such findings provide a framework to bridge disability-related inequalities that characterize the rural-urban divide.

## Conclusions

In conclusion, we found that the prevalence of disability in a rural community in Bangladesh is high. With increasing frequency of hypertension and type 2 diabetes, there is likely to be an increased prevalence of disability. Public health programs to support people from low SES backgrounds, of older age, and female participants in order to bridge disability-related inequities.
